# Supplementary material for: The Systems Biology Research Tool: evolvable open-source software
Source: BMC Syst Biol. 2008 Jun 29;2:55. doi: 10.1186/1752-0509-2-55 (PMC2446383; doi:10.1186/1752-0509-2-55)
Supplement: Additional file 1 — SBRT Archive. An archive of the current version of the Systems Biology Research Tool. [file 1752-0509-2-55-S1.zip › sbrt-1.4.0/doc/developers_guide/api/sbrt/shell/text/TrueFalseFormat.html]

TrueFalseFormat


|  |  |  |
| --- | --- | --- |
| **Overview** \| **Package** \| **Class** \| **Use** \| **Tree** \| **Deprecated** \| **Index** \| **Help** | |  |
| **PREV CLASS**   **NEXT CLASS** | **FRAMES**    **NO FRAMES**     **All Classes** |  |
| SUMMARY: NESTED \| FIELD \| CONSTR \| METHOD | DETAIL: FIELD \| CONSTR \| METHOD |  |


---


## sbrt.shell.text Class TrueFalseFormat

```
java.lang.Object
  sbrt.shell.text.TrueFalseFormat
```

**All Implemented Interfaces:**: BooleanFormat, Format, Formatter<java.lang.Boolean>, Parser<java.lang.Boolean>, SimpleFormat<java.lang.Boolean>

---

``` public final class TrueFalseFormat extends java.lang.Object implements BooleanFormat ```

This class is used to define a format for true/false values.
The string
"true" is considered here equivalent to the boolean value
true, and "false" is considered here equivalent to the
boolean value false. Lower and upper case characters are treated as
equivalent.

**Author:**
:   This class was written and documented by
    Jeremiah Wright while in the Wager lab.

---

**Constructor Summary**

`TrueFalseFormat()`
  
          Constructs a new true/false format object.


| **Method Summary** | |
| --- | --- |
| `java.lang.String` | `format(boolean b)`             Returns "true" or "false" depending on the provided boolean value. |
| `java.lang.String` | `format(java.lang.Boolean b)`             Returns "true" or "false" depending on the provided boolean value. |
| `java.lang.Boolean` | `parse(java.lang.String b)`             Parses the provided string and returns its corresponding boolean value. |
| `boolean` | `parsePrimitive(java.lang.String b)`             Parses the provided string and returns its corresponding boolean value. |

**Methods inherited from class java.lang.Object**

`clone, equals, finalize, getClass, hashCode, notify, notifyAll, toString, wait, wait, wait`

**Constructor Detail**

### TrueFalseFormat

```
public TrueFalseFormat()
```

:   Constructs a new true/false format object.


**Method Detail**

### format

```
public java.lang.String format(java.lang.Boolean b)
```

:   Returns "true" or "false" depending on the provided
    boolean value.

    :   **Specified by:**: `format` in interface `BooleanFormat` **Specified by:**: `format` in interface `Formatter<java.lang.Boolean>`
    :   **Parameters:**: `b` - the boolean value to be formatted. **Returns:**: "true" if `b` is `true`; "false" if `b` is `false`.

---


### format

```
public java.lang.String format(boolean b)
```

:   Returns "true" or "false" depending on the provided
    boolean value.

    :   **Specified by:**: `format` in interface `BooleanFormat`
    :   **Parameters:**: `b` - the boolean value to be formatted. **Returns:**: "true" if `b` is `true`; "false" if `b` is `false`.

---


### parse

```
public java.lang.Boolean parse(java.lang.String b)
```

:   Parses the provided string and returns its
    corresponding boolean value. The string is first
    stripped of leading and trailing whitespace and
    then compared to the strings "true" and "false".
    The case is ignored during this comparison.

    :   **Specified by:**: `parse` in interface `BooleanFormat` **Specified by:**: `parse` in interface `Parser<java.lang.Boolean>`
    :   **Parameters:**: `b` - the string to be parsed. **Returns:**: `true` if `b` matches "true"; `false` if `b` matches "false". **Throws:**: `FormatException` - if the provided string does not match "true" or "false".

---


### parsePrimitive

```
public boolean parsePrimitive(java.lang.String b)
```

:   Parses the provided string and returns its
    corresponding boolean value. The string is first
    stripped of leading and trailing whitespace and
    then compared to the strings "true" and "false".
    The case is ignored during this comparison.

    :   **Specified by:**: `parsePrimitive` in interface `BooleanFormat`
    :   **Parameters:**: `b` - the string to be parsed. **Returns:**: `true` if `b` matches "true"; `false` if `b` matches "false". **Throws:**: `FormatException` - if the provided string does not match "true" or "false".


---


|  |  |  |
| --- | --- | --- |
| **Overview** \| **Package** \| **Class** \| **Use** \| **Tree** \| **Deprecated** \| **Index** \| **Help** | |  |
| **PREV CLASS**   **NEXT CLASS** | **FRAMES**    **NO FRAMES**     **All Classes** |  |
| SUMMARY: NESTED \| FIELD \| CONSTR \| METHOD | DETAIL: FIELD \| CONSTR \| METHOD |  |


---
